# Practical Observations on Leucorrhœa, Fluor Albus, &c.

**Published:** 1830-10-01

**Authors:** 


PRACTICAL OBSERVATIONS ON LEUCORRHŒA, FLUOR ALBUS, OR WEAKNESS, WITH CASES ILLUSTRATIVE OF A NEW MODE OF TREATMENT. By *George Jewel*, Member of the Royal College of Surgeons, one of the Accoucheurs to the St. George's and St. James's Dispensary, &c. 8vo. 1830.

OUR readers are aware that, in our 24th Number, page 517, et seq. we gave an account of Mr. Jewel's paper on the use of nitrate of silver in leucorrhœa, as published in the Medical and Physical Journal, for October, 1829. The present essay is an amplification of that paper, including some additional experience, and a more systematic investigation of the subject.

The first and second chapters of the present work are dedicated to the pathology of leucorrhœa. The popular opinion, no doubt, is, that leucorrhœa proceeds from debility, whether local or constitutional. This opinion is countenanced at least, if not supported, by medical authorities. Dr. Clark, when speaking of the transparent mucous discharges, unaccompanied by change of structure, classes such affections under two heads—namely, those which originate from, or are accompanied by, increased action in the vessels of the part, and others which arise from debility. Mr. Burns has given an opinion somewhat the same, and so have many other authors.

"A minute pathological inquiry must, I think, lead to the conclusion, that local irritation, determination, or inflammation, is the immediate exciting cause. Whilst some writers have insisted on leucorrhœa being always a local disease, having its seat, for the most part, in the uterus or vagina; others have maintained it to be symptomatic, having its origin in general functional disturbance of the system. To this not by any means unimportant part of the subject, I have directed my attention, in order to discover which opinion was entitled to credit; and I have been led to the belief, that the vaginal discharge is commonly the result of some direct local stimulus. That cases do occur, which seem to depend upon a disordered state of the digestive organs, or disturbance of the general health, is obvious; but this altered or relaxed state of fibre, is one which particularly predisposes to local inflammation, or congestion. BATTIN, however, who has written upon this subject, makes his eighth species of the disease, 'leucorrhœa from indigestion;' whilst Pinel commences his classification with the 'constitutional' variety. It is not unusual for a female to have excessive leucorrhœal discharge accompanied by great disturbance of the system. Vertigo, a preternatural heat of the surface, a coated tongue, thirst, and a full pulse, are the symptoms which often accompany, and sometimes precede, the vaginal discharge; when, after a few days of active purgation, and a strictly vegetable diet, all the symptoms, together with the discharge, entirely disappear. Here it must be evident that the uterine vessels partake of the general plethoric state, or disturbance of the system." 4.

Dr. Dewees has divided vaginal discharges into three classes—one (the leucorrhœa of direct irritation) following inflammation of the mucous membrane of the uterus or vagina, produced by some local cause, as laborious parturition, irritating substances applied to the surface of the vagina, &c. Secondly, the "leucorrhœa of remote irritation," in which are ranged all those instances in which the vagina sympathises with some other organs or structures of the body, as with the uterus during pregnancy, or with the same in long-obstructed menses—with the rectum when irritated by hæmorrhoids, &c. Under the third head (the leucorrhœa of habit) he includes those instances of the discharge, which continue after the active or inflammatory condition of the parts has ceased; as after syphilis or gonorrhœa has been cured, a prolapsed uterus restored, &c. It is evident that Dr. Dewees considers the disease as almost invariably of local origin.

"Every protracted or severe case of leucorrhœa, will be attended by great functional disturbance, an interrupted digestion,* a pallid and leucophlegmatic countenance, scanty and irregular menstrual evacuations, a morbid sensibility of the nervous system, oppressed respiration, and exhaustion of the vital powers. It will produce, in short, a variety of anomalous complaints; not unfrequently a remarkable coldness of the feet and legs, and prolapsus, or falling down of the womb, and sometimes prevents conception from taking place. Such are the common effects of profuse vaginal discharges; and hence will appear the difficulty, in the more advanced period of the disease, of distinguishing between cause and effect. The various dyspeptic and other symptoms, which sometimes prove so distressing to the patient, I conceive are, for the most part, secondary or sympathetic, and consequently, if we succeed in removing the local disease, these will gradually disappear. It would, however, be injudicious to direct the attention wholly to the one, to the neglect of the other. It has been well observed by an able writer, that an idiopathic and organic affection of some part may co-exist with disorder of the general health, and that which was a mere functional complication in the beginning, may become organic disease in the sequel." 7.

As to the actual seat of the disease, much diversity of opinion has, as usual, obtained. Cullen, Leake, Hamilton, Sylvius, Astruc, &c. seem to think the leucorrhœal discharge issues from the same vessels that furnish the menstrual secretion. Mr. Jewel believes that this discharge seldom issues from the uterine cavity. The uterus is no doubt lined by a mucous membrane, and, like all other tissues of that kind, may occasionally throw out a superabundant quantity of mucus, or even pus.

* "'Le derangement des digestions accompagne constamment leucorrhée constitutionelle.'"—Gardien.

"By attending to the following circumstances, we shall, in some cases, be able to ascertain whether the discharge issues from the uterine cavity, or not. When the seat of the disease is in the vagina, or cervix of the uterus, the discharge commonly appears during the night, notwithstanding the patient is confined to the horizontal position, but if in the cavity of the womb, it is generally suspended: a piece of sponge, therefore, being introduced into the vagina at bed-time, will occasionally determine the question; for if the discharge issues from the surface of the vagina, it will become saturated with it. This test, it must be admitted, is not implicitly to be relied on; although, by being often repeated, it may throw considerable light upon the pathology of the disease. The discharge issuing from the cavity of the womb, so completely deranges the functions of this organ, that in almost all cases it renders the female incapable of conception. A purulent discharge, the result of active inflammation in the mucous lining of the uterus unconnected with parturition, is not common in its occurrence.

Some writers, who seem to have devoted a good deal of attention to female diseases, state that most leucorrhœal discharges arise from the mucous surface of the vagina. Dr. DEWEES has declared his belief that it consists in an altered action of the vaginal lacunæ, or glands, which furnish, in a state of health, the moisture so important to the part; and Mr. BURNS imagines that the most ample and most frequent source is from the vagina.

It must be admitted, that a great pathological difficulty sometimes arises, in deciding upon the tissue or part which has been morbidly excited: but it is fortunate, that in the majority of cases, the local habitation of the disease is of limited extent; and I may add with confidence, that we possess a remedy which, if judiciously employed, will generally effect a cure, even under most unfavourable circumstances." 11.

Three states or conditions, Mr. J. observes, are requisite for the production of leucorrhœa—irritation, congestion, inflammation of the subacute or chronic kind. NAUCHE in France, and GOOCH in this country, have separated irritation of the uterine system from inflammation of the same. Mr. Jewel does not quite accord with Dr. Gooch, in his definition of IRRITABLE UTERUS—namely, a painful state of that organ, neither attended by, "*nor tending to produce a change in its structure.*" This, he thinks, is a bold assertion, since unhealthy action, if long kept up in a part, may eventually produce "a decisive morbid change." When we consider how long the most painful affections, for example, neuralgia, will go on without any appreciable alteration of texture, we cannot question Dr. Gooch's definition. Besides, although it is probable that leucorrhœa may be attended with chronic inflammation of the parts, it does not follow that a painful or irritable uterus should be attended by the same. Pain may and does exist, quite independent of inflammation. Mr. Jewel introduces a long extract from Dr. Gooch, on irritable uterus, for the purpose of making a pathological distinction between irritable uterus and leucorrhœa.

"First in reference to the pain experienced by the patient, when pressure is applied over the pelvic region, and the absence of leucorrhœa, as indicative of the body of the uterus being the seat of the disease; and secondly, the presence of a copious vaginal discharge, at first thin and transparent, then becoming thick and opaque, as indicative, either that the morbid action is limited, or has extended, to the cervix uteri.

The sequelæ of this irritable state of the uterus, and its contiguous parts, are great depression of spirits, languor, palpitation of the heart, hysterical and other nervous sensations, in all their Protean shapes." 18.

We shall now introduce a case to shew Mr. Jewel's practice in this troublesome complaint.

"*Case* 1.—S. J. ætat. 49, residing in Bridle Lane, admitted a patient under my care at the St. George's and St. James's Dispensary, on the 10th of June. She is the mother of fourteen children, exclusive of two abortions, and has, during the last twelve months, been subject to profuse catamenia, and excessive leucorrhœal discharge of a yellowish colour. She has pain in the loins, shooting in paroxysms through the region of the uterus, in which there is also a sense of fulness and throbbing. She complains of great languor, with loss of appetite and uneasiness at the pit of the stomach. She is frequently attacked with the globus hystericus, and disturbance about the head, and says that a flow of tears affords her much relief. Pulse 85. Bowels confined.

Ten ounces of blood to be abstracted from over the sacrum by cupping.

℞. Magnes. Sulph. ʒvj.

Infus. Rosæ. . ℥vijss.

Acid Sulph. dil. ʒj.—M. ft. Mist. cujus sumantur Cochlearia duo vel tria ampla, mane, quotidie.

℞. Argent. Nitrat. gr. xij.
Aq. distill. .... ℥vj.—M. ft. Injectio.

14th. During a period of twenty-four hours after the cupping, she felt extremely faint and sick, and now complains of increased languor. Says she has used the injection regularly, notwithstanding the presence of the catamenia, and that it occasioned no degree of pain, except a little smarting, the parts having been for some time in an irritable state.

To omit the aperient medicine.

The strength of the injection to be increased from grs. ij. to grs. iv. to the ounce of water; and to take a pill, containing five grains of the extract of hyosciamus and half a grain of opium, at bed time.

18th. The sanguineous discharge has ceased, having continued only a week, its usual period being from ten days to a fortnight. The leucorrhœal fluid has become 'white and thinner than it has been for several months.' The local pains are greatly relieved, but she still complains of occasional heat and throbbing about the womb.

To continue the injection.

22d. The leucorrhœa has ceased, and the local heat and pains have almost left her. There is still great languor and loss of appetite.

℞. Infus. Rosæ .... . ℥vijss.

Sulph. Quinin. .. ℈j.

Tinct. Card. comp. ℥ss.—M. ft. Mist. cujus sumantur Cochlearia duo ampla ter die.

To continue the injection.

26th. The vaginal discharge has not re-appeared. Her spirits are better, and the appetite improves.

Continuentur remedia.

30th. There is no leucorrhœa. Her general health continues to improve, and she intends to go into the country in the course of a few days. Discharged cured." 21.

This discharge often appears immediately before and after the menstrual period—and only at such times. This, he thinks, may be accounted for on the principle of congestion. A congestive state of the utero-vaginal vessels, however, he generally attributes to irregularities of diet and to sedentary habits. Mr. Jewel confidently avers that, whether the disease under consideration proceed from morbid sensibility, congestion, or irritation of the parts, the nitrate of silver will be equally efficacious in remedying the malady.

"From a strict pathological investigation into the numerous cases of leucorrhœa which have fallen under my observation, I have been induced to believe, that when the morbid secretion is abundant, and the local symptoms severe, one uterine affection gives rise to the disease more frequently than any other, namely, a sub-acute or chronic inflammation of the cervix uteri. Even when the vaginal surface appears to have been the tissue primarily affected, we shall find, in almost all protracted cases, the cervix uteri also seriously involved in the mischief. Mr. Burns, an authority which cannot be quoted without respect, alludes to this part of the subject by observing, 'that when the discharge is very opaque, and attended by considerable pain in the back and loins, there is reason to think that the cervix uteri is in a state of irritation; and by examination may be found tender to the touch, and the mouth soft and enlarged a little. This state does not constitute disease of structure, though it may lead to it, but it consists merely in an affection of the glands. After the tender state is nearly subdued, and the discharge has become more chronic, the cold bath, tonics, and astringent injections are proper.' Here it is evidently meant that, as long as the tender state of the cervix uteri continues, we ought not to employ those means which are usually had recourse to, with a view of giving tone and vigour to the system, which, in fact, is an admission of the inflammatory character of the disease. I have scarcely ever seen an instance of profuse leucorrhœa, without more or less tenderness of the cervix uteri. I have also reason to know that very many of such cases are mistaken for carcinoma uteri, and that, in consequence, no remedies are prescribed, or a very inefficient mode of practice is adopted. It may be difficult, it is true, in some cases, to discriminate between a chronic inflammatory affection of the cervix uteri, and incipient scirrhous disorganization. The following remarks will, probably, assist the young practitioner in his diagnosis :—This inflammation of the cervix uteri, like scirrhus, or any organic disease of the uterine system, attacks occasionally at the period of life when the catamenia are about to cease, but I have more frequently found it to exist in married females, from the age of twenty-six or twenty-seven to that of forty, and I have recently seen several cases occurring in young married females, within three months after the birth of the first child. The local symptoms in both diseases, are very nearly allied. There will be occasional lancinating pains through the region of the uterus, with a constant dull kind of pain about the inferior portion of the sacrum, the hip or groin, attended by an irritable bladder, or frequent desire to void the urine, and, in some severe instances, by tenesmus, and pain within the vagina when in the sitting posture. The vaginal discharge is commonly of a milky or cream-like colour, now and then having a glutinous consistence; and is often, in the more acute cases, mixed with a dark-coloured or grumous secretion. Menstruation, if not interrupted by lactation, may be resumed with its usual regularity, although, after a time, some deviation takes place: generally, in the first instance, by its continuing several days beyond the accustomed period. I have remarked that, although the local pains are not unfrequently increased in severity at the commencement of menstruation, a great relief is afforded as soon as the catamenial secretion becomes more abundant. Upon making an examination per vaginam in this disease, the os uteri will not be found opened to the same extent as in scirrhus, (an exception may be made in the case of a woman who has had a numerous family); nor will its margin present the same cartilaginous hardness to the touch. The pain does not appear to be situated in the edges of the os uteri, as described by some authors, but in the cervix, as pressure upon this part alone occasions the patient to complain. The uterus will be found projecting lower in the vagina than natural, but this will depend upon the nature of the disease; the more acute, the further it will have descended. It should be recollected, that prolapsus uteri is a very common effect of protracted leucorrhœa, when, in addition to the symptoms already enumerated, there will be fulness about the pudendum, or weight on the perinæum, and a dragging sensation about the loins, with difficulty in voiding the urine, and sometimes extreme pain in coitu, whilst the discharge will be frequently tinged with blood. These symptoms become modified or severe, according to the degree of descent which has taken place, or the excitability which exists in other and distant organs; hence, in a case of simple relaxation, there will oftentimes be merely a sensation of weakness, and fulness about the pubes, with an increased, but mild, mucous discharge from the vagina. I have seen several cases of prolapsus uteri, in their incipient state, most effectually relieved by the application of the means hereafter named." 29.

Inflammation of the mucous membrane of the vagina—"an important cause of leucorrhœa"—may be acute or chronic—the latter being the most common and the most insidious. One of the first symptoms, he observes, is often an increased secretion of thin mucus, unaccompanied by pain or irritation. The progress, however, of the disease, is marked by a sense of itching or burning, with occasional swelling about the labia. As the complaint advances, the vaginal secretion changes in consistence and appearance, becoming thicker, and staining the linen of a yellow colour—or assuming a brownish hue, when it is usually offensive. The glands of the groin seldom enlarge, nor is there usually much ardor urinæ, unless the discharge be extremely acrid, the vaginal surface highly sensible, or excoriation exist. After the subsidence of these symptoms, which may continue for a longer or shorter period, the patient complains of languor and debility—looks pale, with want of animation in the countenance, and a dark semicircular appearance under the eyes. The digestive functions become much disturbed, and some degree of uneasiness with distention is usually complained of in the stomach.

"Inflammation of the mucous surface of the vagina may arise from any of those causes which produce the same action in other textures, and which may be classed under two heads:—general and local excitement. The circumstances which induce the former state, may be considered as follows; namely, a high nutritious diet, and the free use of wines, spirituous or fermented liquors; violent exertions of the body, such as dancing; pyrexia, or fever, and exposure to cold. In short, such complaints may arise from any unnatural activity in the vascular or nervous systems. Among the various local causes, may be noticed, difficult parturition, blows, the lodgement of extraneous bodies, as a pessary or piece of sponge, too frequent coition, &c.; whilst misplacements of the uterus, or its various diseases, such as scirrhus, hydatids, polypus, &c. will commonly produce irritation and inflammation, with a discharge of unhealthy mucus from the vagina. Local excitement, either in the vagina, or neck of the womb, or both, is often produced by hemorrhage, or abortions. Painful menstruation, in which there is commonly congestion of the uterine vessels, will occasionally be preceded and followed by an increased secretion from the mucous membrane of the vagina." 38.

M. Blattin examined the bodies of 24 females who died from excessive leucorrhœal discharges, with the view of ascertaining the seat of the disease. In nine of these cases, the morbid secretion was found to arise from the uterus—in thirteen, from the neck of the uterus and vagina—and in two, from the fallopian tubes. A mucous discharge is not unfrequently discovered to arise from excoriations about the nymphæ—a species of the disease purely local, and for which Mr. Jewel thinks the nitrate of silver peculiarly adapted.

This brings us to the 3d chapter of the work, treating of leucorrhœa in children—in pregnant women—and at the "turn of life."

Mucous and muco-purulent discharges are very apt to occur in children, in consequence of dentition, want of cleanliness, exposure to cold, &c. attended with ardor urinæ, inflammation, excoriation, and other troublesome symptoms. Again, in females about the age of puberty, the regular action of the uterus is prevented by leucorrhœa.

"In the treatment of vaginal discharges in children, the strictest cleanliness ought to be observed, as the secretions so soon become acrid. The parts should therefore be washed carefully with a little tepid vinegar and water, (or if this wash should occasion pain, milk and water may be substituted,) and wiped perfectly dry, at least twice a day. It often happens in young children, as well as in females arrived at the age of puberty, that there is a preternatural fulness in the system, indicated by head-ache, thirst, and a quick hard pulse; in which case, active purging, or blood-letting, should be premised, to diminish arterial action, before the nitrate of silver, or other local remedies are employed. It is only necessary to add, that the uterus seldom takes on its regular and healthy function, until the leucorrhœa be removed: an object which, in most instances, may be accomplished, by attention to the rules laid down in another part of this work." 43.

From the many instances of this discharge which we have seen in female children, and all readily giving way to cleanliness and common astringent injections of the sulphate of zinc, we cannot but consider the use of the nitrate of silver as, at least, unnecessary in nine cases out of ten of such affections. Mr. Jewel himself cautions us not to be too free with the nitrate in the leucorrhœa of utero-gestation. It has been remarked by Denman, that those females who suffer most from this complaint, have easiest labours. Our author saw one remarkable illustration of this maxim, but is not prepared to say that the rule is absolute. In general there is a state of local repletion induced by pregnancy, and Mr. Jewel thinks that, as the leucorrhœal discharge acts as a depletion on the internal organs, it ought not to be suppressed until the congestive state of the uterine system be first removed. One thing ought to be attended to strictly—frequent ablutions during labour to prevent the infant from being affected by leucorrhœal ophthalmia.

In respect to the profuse leucorrhœal discharge which sometimes occurs about the cessation of the catamenia, it must be looked upon with suspicion of structural disease. To secure a female from those ailments which occasionally shew themselves about the TURN OF LIFE, the diet should be regulated, and exercise enjoined. The usual obesity which occurs about this period renders females little inclined to corporeal exertion. Depletion is often necessary at this critical juncture, to relieve the plethora of the venous system.

"There is one symptom, the effect, in most instances, of the acrimonious quality of the discharge, which is oftentimes excessively harrassing, namely, pruritus, or itching of the parts; and it is one which demands the attention of the practitioner, inasmuch as it not unfrequently indicates disease of the organs within, as of the uterus, bladder, &c. It appears to arise from the lodgement of the irritating secretion in the vulva, or vagina. I am of opinion, that pruritus rarely comes on, without there being an acrid vaginal secretion, or an efflorescence upon the internal surface of the parts, although it may be so trifling as to escape the observation of the patient herself. A lady who had laboured under incessant pruritus, with a slight leucorrhœal discharge, for a period of four months, was completely restored, by injections of the solution of the nitrate of silver. Dr. Dewees, by whom the aphthous efflorescence above alluded to was first brought to the notice of the profession, recommends, in strong terms, for the cure, a solution of borax in water, both as a wash and as an injection. An injection of the liq. ammoniæ puræ, in the proportion of a tea-spoonful to a pint of water, has been found useful; but I would place the greatest reliance upon injections of the solution of the nitrate of silver. Blood-letting, local or general, together with a low vegetable diet, will be essentially necessary to the cure of the complaint, although I have seen more than one obstinate case yield, in the course of a few days, to the injection last named, without any other aid." 56.

The 5th chapter of the work treats of the predisposing and exciting causes—the influence of seasons—of a contaminated atmosphere, &c.

Among the most common of the predisposing and exciting causes, Mr. Jewel reckons a scrofulous diathesis—irritable nervous system—derangement of the menstrual secretion—frequent parturitions or abortions—protracted lactation, &c. Mr. J. accords with Leake and some others, that leucorrhœa prevails more in the Autumn than in any other season of the year. Moisture and cold unquestionably predispose to the disease. The impure atmosphere of large cities and crowded apartments conduces much to the propagation of the malady.

The sixth and last chapter is on the treatment of leucorrhœa. Our author, as may have been anticipated, "places the most perfect reliance on the nitrate of silver," while he is anxious to impress on the attention of practitioners, the necessity, in all cases, of adopting what may be termed general principles. The state of the general health, therefore, and that of the circulation, should be examined into. Upon these points we need not dwell. Mr. Jewel thinks that more harm than good is done by cold sponging the loins, so often had recourse to, with or without advice. He prefers a tepid ablution consisting of one part of vinegar to two of water. The cold bath is often hazardous, where the patient is enfeebled by the discharge, easily put out of breath, and whose digestive organs are disordered. Of the lytta Mr. Jewel does not seem to entertain much opinion. Astringent injections, he observes, have very generally failed to cure this complaint.

"There is one medicine which hitherto has been employed upon a very limited scale in leucorrhœal diseases, but which, I have every reason to believe, is peculiarly adapted to assist in their removal. I allude to iodine. The efficacy of iodine over the absorbent system is now so completely established, as scarcely to require further comment, indeed, it may be stated, without fear of contradiction, that we have no article in the materia medica, possessing more influence over, or so capable of producing such extraordinary and important changes in the glandular parts of the body, as this medicine. Its effects upon the uterine system in particular, in almost all the cases in which I have employed it, have been marked and decisive.

I may here notice a case of diseased ovary, in which this little, but important, organ had morbidly increased to the size of the fœtal head. The general and visceral disturbance occasioned by its presence in the pelvic cavity, had become so distressing, that the patient, notwithstanding the fatality of an operation had been represented to her, often expressed an earnest desire to have it removed. After various means had been employed, without any beneficial result, she was put upon a course of iodine, commencing with ten drops of the tincture three times a day, gradually increasing the dose to thirty-five. She has been under the influence of this medicine about ten weeks, and at the present time, the tumour is scarcely to be felt. She has suffered nothing from such large doses of the medicine, but, on the contrary, her spirits are greatly improved, and she anticipates, with great confidence, a perfect restoration to health. Dr. Thomson, the able professor of materia medica at the London University, has related a case of ovarian dropsy, in which, after the woman had been tapped in the usual manner, and seven quarts of albuminous serum, mixed with pus, removed, iodine was administered, and carried to the extent of thirty-six drops of the tincture three times a day. The result was, that the tumour wholly disappeared, and the woman was perfectly restored." 80.

The mode of applying the nitrate of silver is detailed at length in our 24th No. p. 517, and therefore we need not reprint it here.

Under the head of gonorrhœa, Mr. Jewel informs us that the nitrate of silver is as effectual in that complaint as in leucorrhœa. The use of this remedy is not new in gonorrhœa, as it was very commonly employed, to our knowledge, by our naval surgeons during the late war. In females we find that Mr. Jewel employs the nitrate in the proportion of three grains to the ounce of water.

We return Mr. Jewel many thanks for the perusal of his little volume, which contains much judicious observation, with the open and liberal communication to his brethren of a remedy which promises to be of considerable service in a troublesome and obstinate complaint.

---

